# Frequency of Pre-obesity and Obesity in Medical Students of Karachi and the Predisposing Lifestyle Habits

**DOI:** 10.7759/cureus.3948

**Published:** 2019-01-23

**Authors:** Ayesha Asghar, Aresha Masood Shah, Abbas Ali Hussain, Amber Tahir, Hajra Asghar

**Affiliations:** 1 Internal Medicine, Jinnah Sindh Medical University, Karachi, PAK; 2 Internal Medicine, Dow University of Health Sciences, Karachi, PAK; 3 Dentistry, Dow University of Health Sciences, Karachi, PAK

**Keywords:** overweight, pre-obesity, obesity, medical students, food habits, physical activity, karachi, pakistan

## Abstract

Background: The global incidence of pre-obesity and obesity is rising noticeably. Where medical students should be advocating healthy lifestyles, they are actually indulging in unhealthy eating habits and sedentary lifestyle. This is resulting in an increased incidence of obesity in this population.

Methods: It was a descriptive cross-sectional study conducted from September 2018 till January 2019 in four medical colleges of Karachi. Sociodemographic profile, body mass index (BMI), food habits, and exercise routine of the students were recorded. Data was entered and analyzed using SPSS version 22 (IBM Corp., Armonk, NY).

Results: The mean BMI of the study population was 21.717 ± 4.33 kg/m^2 ^(range: 16.24–38.19). The mean age was 21.345 ± 1.4709 years with a minimum of 17 years and maximum of 25 years. The combined frequency of pre-obese and obese students was 33.2%. Among these, there were more women (60.6%) than men (39.3%). Among these pre-obese and obese students, 55% had breakfast rarely to sometimes, 47.9% took four or more meals per day, 39.3% consumed fast food thrice weekly or more, and 58.1% consumed soft-drinks/juices alternate to every day. Among the students who indulged into binge eating when stressed, 56.9% were pre-obese to obese. The nutritional status of the study sample was significantly associated with female gender, living status with parents, irregular breakfast, infrequent daily meals, increased consumption of fast food and beverages, decreased consumption of red meat, sedentary lifestyle, and altered eating habits when stressed.

Conclusion: The incidence of pre-obesity and obesity is noticeably escalating among young adults. If this trend continues, obesity-related complications will form the major chunk of medical illnesses in the near future. Strategies are needed to nip this dilemma in the bud by indulging in healthy and clean eating habits and performing regular physical activity.

## Introduction

Not a problem of a certain part of the world any longer; obesity has now become a global pandemic. The number of individuals falling prey to this health hazard is multiplying dramatically. In 2016, more than 1.9 billion individuals of age 18 and above were pre-obese and around 650 million of them were obese [1]. if the current trends continue to rise in the similar pattern, it is expected that by 2030, 2.16 billion individuals (38%) will be pre-obese and 1.12 billion (20%) will be obese [2].

Where genetic predisposition cannot be neglected as a contributing factor to obesity; diet, physical activities, and behavioral factors are also crucial risk factors [3]. Morbid obesity complicates into cardiovascular diseases, metabolic disturbances including diabetes mellitus and polycystic ovarian disease, gastroesophageal reflux, and certain types of malignancies [4]. It has been established that around 44% of the diabetes mellitus burden and 23% of the cardiovascular diseases burden is contributed by pre-obesity and obesity. Every year 2.8 million adult individuals die due to obesity and its complications [5].

In a large cohort, 19.4% of Asians had a body mass index (BMI) of 25 or more. When subcategorized, it was established that 23.9% of South Asians had their BMI of 25 or more. Sixty-seven percent of these South Asians were men, and 33% were women [6].

By default, healthcare providers and facilitators should be presenting themselves as advocates and ambassadors of healthy lifestyle for the general population. However, studies suggest otherwise. Scientific evidence is present which endorse unhealthy eating habits [7-8], disturbed nutrition status [9], lack of physical activity [10], and low quality of life [11] among these healthcare activists.

Increased intake of high-calorie foods and a sedentary life have tripled the rate of obesity among the young adults of the developing countries over the last 20 years. Several studies among university students in multiple developing countries including India, Bangladesh, and China suggested a high prevalence of obesity [12].

In a recent study from Lahore, 21% of medical students were of BMI 25 or more. There were 46% men with central obesity and 31.4% were women. The associated risk factors were high-calorie food, studying at private medical colleges, male gender, lack of sports, and no regular exercise [3]. This study emphasizes on assessing the nutritional status of the medical students and compare it with their dietary habits and physical activities.

## Materials and methods

It was a descriptive cross-sectional survey-based study which was conducted in four medical colleges of Karachi. Two public medical colleges and two private medical colleges were included. The maximum combined strength of these colleges is 4,000 students. With an anticipated frequency of 50% and a confidence interval of 95%, the sample size calculated was 351. Non-probability convenient sampling technique was adapted and data was collected from September 1, 2018 till January 31, 2019.

Sociodemographic profile of the students was recorded. It included their age, gender, medical college name, year of education, and their living status (living with parents/hostel). Body weight (in kilograms) and body height (in meters) was recorded to calculate their BMI. BMI was categorized as per the standard World Health Organization (WHO) classification [13]. Responses regarding their food habits and exercise routine were recorded. Data was entered and analyzed using SPSS version 22 (IBM Corp., Armonk, NY). Mean and standard deviation (SD) were calculated for continuous variables. Frequencies and percentages were calculated for incontinuous variables. Correlations were established using Chi-square (p<0.05 was taken significant).

## Results

Almost three-fourth of the study sample comprised of female students. The mean ± SD age of the study sample was 21.345 ± 1.4709 years with a minimum of 17 years and maximum of 25 years. The sociodemographic profile of the students is shown in Table 1.

**Table 1:** Sociodemographic profile of the participating students (N=351)

Sociodemographic variable	Frequency (%)
Gender	
Male	93 (26.5)
Female	258 (73.5)
Year of education	
First year	66 (18.8)
Second year	81 (23.0)
Third year	78 (22.2)
Fourth year	82 (23.4)
Final year	44 (12.5)
Living status	
With parents	267 (76)
At hostel	84 (24)
Medical college	
Public	170 (48.5)
Private	181 (51.5)
Family history	
Obesity	124 (35.3)
Diabetes mellitus	224 (63.8)
Hypertension	198 (56.4)
Ischemic heart disease	204 (58.1)
Age in years (Mean ± SD)	21.345 ± 1.4709

The mean ± SD BMI of the study population was 21.717 ± 4.33 kg/m2 (range: 16.24–38.19). There were 93 students (26.5%) with BMI < 18.5 (underweight), 141 students (40.2%) with BMI between 18.5 and 24.9 (normal weight), 52 students (14.8%) with BMI between 25 and 29.9 (pre-obesity), 42 students (11.9%) with BMI between 30.0 and 34.9 (obesity class I), and 23 students (6.5%) with BMI between 35.0 and 39.9 (obesity class II).

None of the students were beyond BMI 39.9. The pre-obese, obese I, and obese II students (33.2%) were classified according to their sociodemographic profiles as shown in Table 2.

**Table 2:** Gender, medical college, and living status of pre-obese and obese medical students (N=117) * p <0.05, Chi-square applied

	Gender		Medical college		Living status	
Nutritional status (BMI ≥ 25; N=117)	Male (N=93)	Female (N=258)	Public (N=170)	Private (N=181)	With parents (N=267)	Hostel (N=84)
Pre-obese (n=52)	20/93 (21.5%)	32/258* (12.4%)	24/52 (46.2%)	28/52 (53.8%)	47/52* (90.3%)	5/52 (9.7%)
Obese I (n=42)	18/93 (19.3%)	24/258* (9.3%)	16/42 (38%)	26/42 (62%)	38/42* (90.4%)	4/42 (9.6%)
Obese II (n=23)	8/93 (8.6%)	15/258 (5.8%)	15/23 (65.2%)	8/23 (34.8%)	19/23 (82.6%)	4/23 (17.4%)
Total	46/93 (49.4%)	71/258 (27.5%)	55/117 (47.1%)	62/117 (52.9%)	104/117 (88.9%)	13/117 (11.1%)

Only 62 of 351 (17.7%) students played sports at college; 45 of these were male students (72.5%). The median sitting time of the students for studying purposes (studying at university, home and/or library) was 7.4 hours (range 6–12 hours). The median sitting time of the students for non-studying purposes was 3.1 hours (range 1.5–5.5 hours). Only 98 students (27.9%) had a regular habit of standing/strolling while studying. There were 168 out of 351 students (47.8%) who exercised regularly. Their mean time of exercise was 27.15 ± 11.258 min (range: 15–120 min). Only 45 of these 168 students (26.7%) were enrolled at a gym and the remaining did exercise at home.

Among 117 pre-obese and obese students, 65 of them (55%) were taking breakfast rarely to sometimes, 56 (47.9%) were taking four or more meals per day, 46 (39.3%) were consuming fast food thrice weekly or more than that, 68 (58.1%) were consuming soft drinks/juices alternate to everyday, 79 (67.5%) were consuming red meat thrice or more than that weekly. Among 109 students who indulged into binge eating when stressed, 62 (56.9%) were pre-obese to obese. Among 100 students who rarely ate when stressed, 20 (20%) were pre-obese to obese, and remaining 80 (80%) were underweight to normal healthy. The food and drinks habits, stress eating habits, and exercise routine of the students were recorded. Their correlation with the nutritional status of the students is shown in Table 3.

**Table 3:** Food and exercise habits of the students and their corresponding nutritional status

Food and exercise habits	Underweight (N=93)	Normal (N=141)	Pre-obesity (N=52)	Obese I (N=42)	Obese II (N=23)
How often do you eat breakfast?*					
Rarely	18 (19.4%)	01 (0.7%)	03 (5.7%)	19 (45.2%)	18 (78.2%)
Sometimes	20 (21.5%)	31 (22%)	11 (2.1%)	13 (30.9%)	01 (4.3%)
Alternate days	08 (8.6%)	18 (12.8%)	08 (15.4%)	03 (7.1%)	03 (13%)
Every day	47 (50.5%)	91 (64.5%)	30 (57.7%)	07 (16.6%)	01 (4.3%)
How many meals do you take in a day?*					
2 times	36 (38.7%)	67 (47.5%)	16 (30.7%)	03 (7.1%)	01 (4.3%)
3times	48 (51.6%)	54 (38.3%)	27 (52%)	10 (23.8%)	04 (17.3%)
4 times or more	09 (9.6%)	20 (14.2%)	09 (17.3%)	29 (69%)	18 (78.2%)
How much fast food do you consume in a week?*					
None	07 (7.5%)	16 (11.3%)	06 (11.5%)	01 (2.4%)	01 (4.3%)
Once a week	38 (40.8%)	84 (59.5%)	23 (44.2%)	01 (2.4%)	01 (4.3%)
Twice a week	13 (13.9%)	22 (15.6%)	06 (11.5%)	22 (52.3%)	10 (43.5%)
Thrice a week	27 (29%)	13 (9.2%)	14 (26.9%)	12 (28.5%)	09 (39.1%)
More	08 (8.6%)	06 (4.2%)	03 (5.7%)	06 (14.2%)	02 (8.7%)
How often do you consume soft drinks / juices / energy drinks?*					
Rarely	23 (24.7%)	55 (39%)	18 (24.6%)	04 (9.5%)	01 (4.3%)
Sometimes	39 (41.9%)	38 (27%)	22 (42.3%)	03 (7.1%)	01 (4.3%)
Alternate days	16 (17.2%)	36 (25.5%)	04 (7.7%)	23 (54.8%)	03 (13%)
Every day	15 (16.1%)	12 (8.5%)	08 (15.4%)	12 (28.5%)	18 (78.2%)
How frequently do you consume red meat?*					
Once a week	53 (57%)	58 (41.1%)	08 (15.4%)	07 (16.6%)	01 (4.3%)
Twice a week	17 (18.3%)	33 (23.4%)	12 (23%)	06 (14.2%)	04 (17.3%)
Thrice a week	17 (18.3%)	35 (24.8%)	25 (48%)	22 (52.3%)	09 (39.1%)
More	06 (6.4%)	15 (10.6%)	07 (13.5%)	07 (16.6%)	09 (39.1%)
Do you perform exercise regularly?*					
No	63 (67.7%)	43 (30.5%)	20 (28.5%)	37 (88%)	20 (86.9%)
Yes	30 (32.2%)	98 (69.5%)	32 (61.5%)	05 (12%)	03 (13%)
How is your eating pattern affected when stressed out?*					
Not affected	29 (31.2%)	78 (55.3%)	22 (42.3%)	11 (26.2%)	02 (8.6%)
Binge eat when stressed	24 (25.8%)	23 (16.3%)	16 (30.7%)	26 (62%)	20 (86.9%)
Rarely eat when stressed	40 (43%)	40 (28.4%)	14 (26.9%)	05 (12%)	01 (4.3%)

## Discussion

This study reports a frequency of 33.2% of pre-obesity and obesity among the medical students of Karachi. This nutritional status was significantly associated with female gender and living status with parents. Unhealthy eating habits including irregular breakfast, infrequent daily meals, increased consumption of fast food and beverages, and decreased consumption of red meat were significantly associated with the nutritional status of these students. Sedentary lifestyle and binge eating/rarely eating when stressed were also significant correlators. Medical college status – private versus public – did not show any significant effect on the nutritional status.

It was a multicenter study, with a large sample. It compared the nutritional status of public and private medical college students. It assessed the eating habits of the students in detail including stress eating. However, the study neither calculated the daily calorie consumption nor the calorie expenditure. This balance plays a crucial role in weight gain.

In the past, there have been studies to assess the nutritional status of medical students. In 2012, a Karachi based study reported a combined incidence of 27.1% of pre-obesity and obesity which was significantly associated with male gender [14]. In another study, 21% of medical students were reported to have BMI of 25 or more [3]. However, a recent study from another private medical college in Karachi reported a startling incidence of 63% pre-obesity and 15.4% obesity, although their mean BMI was 23.37±4.63 kg/m2 which is comparable to the BMI reported in this study. They reported significant correlation of nutritional status with male gender [15]. On the contrary, upon stratification, this study deduced a statistically significant correlation of nutritional status with the female gender. This association can be attributed to the fact that this study had three times more women than men. They also reported a higher incidence of pre-obesity and obesity in hostel-dwelling students [15], which is also contrary to this study’s results. Hostel students have very diverse eating habits mainly governed by cooking skills, availability of cooking resources, financial constraints, and frequency of examination [16]. Their poor dietary habits render them at the risk of being at both nutritional extremes. In an Indian study, only 17.5% of hostel residing medical students were reported to be overweight and 3.4% to be obese [17]. 

In another Indian study with 21% pre-obese and 3% obese medical students, 62% were physically active [10]. A study from Pakistan reported that private medical college students were more into strenuous exercises such as swimming and aerobics and public medical college students only indulged in lighter exercises such as walking or climbing stairs. Overall, medical students had low physical wellness [18]. Although the current study did not utilize any standard instrument to assess the status of physical activity, 47.8% reported that they exercised regularly. In another recent study, 43.1% were active to moderately active. Their mean ± SD BMI was 23.59 ± 4.54 (range: 11.24–38.36) [19].

Healthy eating habits and regular physical activity plays a fundamental role in the prevention of obesity and its consequent health-related complications including metabolic and cardiovascular disorders. The rising incidence of “above healthy BMI” in young adults is a global public health concern. These individuals will increase the future burden of non-communicable diseases including dyslipidemia, hypertension, cardiovascular morbidity, subfertility, and others. Hence, preventive strategies are the need of the time. Preventive studies and awareness programs must be conducted on a national scale to highlight this crisis our young generation is going through.

## Conclusions

The incidence of pre-obesity and obesity is noticeably escalating in the young adult population. At this rate, obesity-related complications will form the major chunk of medical illnesses in the near future. Obesity not only reduces the quality of life but also the lifespan. Strategies are needed to nip this dilemma in the bud, particularly among the healthcare personnel who themselves must be practicing and advocating healthy lifestyles. Students must indulge in healthy and clean eating habits and perform regular physical activity.

## References

[1] (2019). World Health Organization: obesity and overweight. http://www.who.int/en/news-room/fact-sheets/detail/obesity-and-overweight.

[2] Kelly T, Yang W, Chen CS, Reynolds K, He J (2008). Global burden of obesity in 2005 and projections to 2030. Int J Obes.

[3] Khan ZN, Assir MZ, Shafiq M, Chaudhary AE, Jabeen A (2016). High prevalence of preobesity and obesity among medical students of Lahore and its relation with dietary habits and physical activity. Indian J Endocrinol Metab.

[4] Abdelaal M, le Roux CW, Docherty NG (2017). Morbidity and mortality associated with obesity. Annals Transl Med.

[5] Misra A, Shrivastava U (2013). Obesity and dyslipidemia in South Asians. Nutrients.

[6] Klatsky AL, Zhang J, Udaltsova N, Li Y, Tran HN (2017). Body mass index and mortality in a very large cohort: is it really healthier to be overweight?. Perm J.

[7] Al-Qahtani MH (2016). Dietary habits of Saudi medical students at University of Dammam. Int J Health Sci.

[8] Ackuaku-Dogbe EM, Abaidoo B (2014). Breakfast eating habits among medical students. Ghana Med J.

[9] Sirang Z, Bashir HH, Jalil B (2013). Weight patterns and perceptions among female university students of Karachi: a cross sectional study. BMC Public Health.

[10] Rao CR, Darshan BB, Das N, Rajan V, Bhogun M, Gupta A (2012). Practice of physical activity among future doctors: a cross sectional analysis. Int J Pre Med.

[11] Pagnin D, De Queiroz V (2015). Comparison of quality of life between medical students and young general populations. Educ Health.

[12] Peltzer K, Pengpid S, Samuels T (2014). Prevalence of overweight/obesity and its associated factors among university students from 22 countries. Int J Environ Res Public Health.

[13] (2019). Body mass index - BMI. http://www.euro.who.int/en/health-topics/disease-prevention/nutrition/a-healthy-lifestyle/body-mass-index-bmi.

[14] Mahmood S, Perveen T, Najjad M, Yousuf N, Ahmed F, Ali N (2013). Overweight and obesity among medical students of public sector’s institutes in Karachi, Pakistan. J Obes Wt Loss Ther.

[15] Jamshed S, Khan F, Bashir A, Abid K, Baig NN (2018). Evaluation of body mass index among undergraduate medical students. Pak J Surg.

[16] Kabir A, Miah S, Islam A (2018). Factors influencing eating behavior and dietary intake among resident students in a public university in Bangladesh: a qualitative study. PloS one.

[17] Gupta S, Ray TG, Saha I (2009). Overweight, obesity and influence of stress on body weight among undergraduate medical students. Indian J Community Med.

[18] Khan R, Rehman R, Baig M, Hussain M, Khan M, Syed F (2015). Dimensions of physical wellness among medical students of public and private medical colleges in Pakistan. Saudi Med J.

[19] Bareeqa SB, Ahmed SI, Samar SS, Allahyar Allahyar, Shera MT (2018). Assessment of physical activity among young students of different institutes; a multicenter experience from a developing country. JOJ Pub Health.

